# Diagnostic Accuracy of Routinely Available Biomarkers to Predict Bacteremia in Children With Community-Acquired Pneumonia: A Secondary Analysis of the GPIP/ACTIV Pneumonia Study in France, 2009–2018

**DOI:** 10.3389/fped.2021.684628

**Published:** 2021-10-21

**Authors:** Danaé Dudognon, Corinne Levy, Martin Chalumeau, Sandra Biscardi, Marie-Aliette Dommergues, François Dubos, Karine Levieux, Marie Aurel, Philippe Minodier, Ferielle Zenkhri, Ellia Mezgueldi, Irina Craiu, Laurence Morin, Stéphane Béchet, Emmanuelle Varon, Robert Cohen, Jérémie F. Cohen, The Pneumonia Study Group, François Angoulvant

**Affiliations:** ^1^Department of General Pediatrics and Pediatric Infectious Diseases, AP-HP, Hôpital Necker-Enfants Malades, Université de Paris, Paris, France; ^2^Association Clinique et Thérapeutique Infantile du Val de Marne (ACTIV), Créteil, France; ^3^Groupe de Pathologie Infectieuse Pédiatrique (GPIP), Paris, France; ^4^Clinical Research Centre, Centre Hospitalier Intercommunal de Créteil, Créteil, France; ^5^Paris Est University, IMRB-GRC GEMINI, Créteil, France; ^6^Epidemiology and Statistics Research Centre - CRESS, INSERM, Obstetrical, Perinatal and Pediatric Epidemiology Research Team, Université de Paris, Paris, France; ^7^Pediatric Emergency Department, Centre Hospitalier Intercommunal de Créteil, Créteil, France; ^8^Department of General Pediatrics, Centre Hospitalier de Versailles-Le Chesnay, Versailles, France; ^9^Pediatric Emergency Unit and Infectious Diseases, Univ. Lille, CHU Lille, Lille, France; ^10^Department of Pediatrics, Centre Hospitalier Universitaire de Nantes, Nantes, France; ^11^Department of General Pediatrics, AP-HP, Hôpital Robert Debré, Université de Paris, Paris, France; ^12^Pediatric Emergency Department, Centre Hospitalier Universitaire Nord, Marseille, France; ^13^Pediatric Emergency Department, AP-HP, Hôpital Le Kremlin-Bicêtre, Université Paris Sud, Bicêtre, France; ^14^Pediatric Emergency Department, Hospices Civils de Lyon, Hôpital Femme-Mère-Enfant, Lyon, France; ^15^Pediatric Emergency Department, AP-HP, Hôpital Robert Debré, Université de Paris, Paris, France; ^16^Centre National de Référence des Pneumocoques, Centre Hospitalier Intercommunal de Créteil, Créteil, France; ^17^Service des Petits Nourrissons, Centre Hospitalier Intercommunal de Créteil, Créteil, France

**Keywords:** pneumonia, bacteremia, biomarkers, procalcitonin (PCT), pneumococcus (*Streptococcus pneumoniae*), diagnostic test, sensitivity and specificity, blood culture

## Abstract

**Objective(s):** Blood cultures (BC), when performed in children seen in the emergency department with community-acquired pneumonia (CAP), are most of the time sterile. We described the diagnostic accuracy of white blood cells (WBC), absolute neutrophils count (ANC), C-reactive protein (CRP), and procalcitonin (PCT) to predict blood culture (BC) result in childhood CAP.

**Study Design:** Secondary analysis of a prospective study carried out in eight pediatric emergency departments (France, 2009–2018), including children (≤15 years) with CAP. Analyses involved univariate comparisons and ROC curves.

**Results:** We included 13,752 children with CAP. BC was positive in 137 (3.6%) of the 3,829 children (mean age 3.7 years) in whom it was performed, mostly with *Streptococcus pneumoniae* (*n* = 107). In children with bacteremia, ANC, CRP and PCT levels were higher (median 12,256 vs. 9,251/mm^3^, 223 vs. 72 mg/L and 8.6 vs. 1.0 ng/mL, respectively; *p* ≤ 0.002), but WBC levels were not. The area under the ROC curve of PCT (0.73 [95%CI 0.64–0.82]) was significantly higher (*p* ≤ 0.01) than that of WBC (0.51 [0.43–0.60]) and of ANC (0.55 [0.46–0.64]), but not than that of CRP (0.66 [0.56–0.76]; *p* = 0.21). CRP and PCT thresholds that provided a sensitivity of at least 90% were 30 mg/L and 0.25 ng/mL, respectively, for a specificity of 25.4 and 23.4%, respectively. CRP and PCT thresholds that provided a specificity of at least 90% were 300 mg/L and 20 ng/mL, respectively, for a sensitivity of 31.3 and 28.9%, respectively.

**Conclusions:** PCT and CRP are the best routinely available predictive biomarkers of bacteremia in childhood CAP.

## Introduction

With 120–150 million new cases each year in the world, including around 14 million severe episodes, childhood community-acquired pneumonia (CAP) is a major public health issue. In 2015, CAP caused about 16% of all deaths in children under 5 years old, which represents 920,136 deaths according to the World Health Organization, with the major burden of the disease being carried by children from low- and middle-income countries (1, 2).

*Streptococcus pneumoniae* and respiratory viruses are the main causative agents of pediatric CAP (3). Clinical features and radiological findings have poor accuracy in discriminating these causes. Also, viral testing may not be sufficient to diagnose viral CAP because of bacterial-viral co-infections that may occur in up to 30% of children with CAP (4). In young children under 10 years, non-invasive microbiological testing such as pneumococcal urinary antigen may indicate carriage rather than true infection (5). Bacteremia is a robust outcome to identify bacterial cases and is also a marker of invasive disease. Blood culture (BC) results are usually available within 24 h. High levels of white blood cells (WBC), absolute neutrophils count (ANC), and C-reactive protein (CRP) are usually considered associated with bacterial disease. However, the diagnostic accuracy of these routinely available biomarkers seems limited (6, 7). Thus, European and North-American guidelines recommend empirical antibiotic treatment based on the assessment of epidemiological factors and clinical severity rather than laboratory and imaging testing (8, 9).

Procalcitonin (PCT) was shown to be a predictor of bacteremia and disease severity in children with CAP (10–12). CRP and PCT are also used as surrogate markers of pneumococcal infection in studies evaluating the effectiveness of pneumococcal conjugate vaccines (PCVs) (13, 14); confirming the association between routinely available biomarkers and bacteremia would further support their use as endpoints in PCV trials. In this large, prospective, multicenter study, we aimed at assessing the performance of four biomarkers (WBC, ANC, CRP, and PCT) to predict bacteremia in a population of children with CAP.

## Materials and Methods

### Study Design and Setting

This is a secondary analysis of a prospective multicenter observational study that was carried out in 8 tertiary pediatric emergency departments across France. The principal study was set up in 2009 to assess the impact of PCV13, which, in France, was implemented in June 2010. Data were collected between June 1, 2009 and May 31, 2018. For each patient, a case report form was completed by a designated senior clinical investigator and sent to the central investigating center (Association Clinique et Thérapeutique Infantile du Val-de-Marne, ACTIV). Following a pragmatic approach, no specific action was taken to standardize patient management across participating centers or among physicians within each center. Notably, indications for chest radiograph, blood tests, criteria for hospital admissions, and antibiotic treatments were left at the discretion of the treating physician. Detailed methods were described in previous reports stemming from the same overarching study (13–16). We followed the STARD 2015 (Standards for Reporting Diagnostic Accuracy Studies) reporting guidelines (Appendix 1) (17). The Robert Debré Hospital Ethics Committee approved the study. The data collection was approved by the French National Data Protection Committee (Commission Nationale de l'Informatique et des Libertés, No. 1348184).

### Study Participants

We included all children aged 1 month to 15 years with CAP, which was defined by the association of fever (body temperature ≥38.5°C for children above 3 months and ≥38°C for children under 3 months) or hypothermia, and a chest radiograph showing consolidation, as diagnosed by the senior pediatrician in charge and confirmed by a pediatric radiologist. Indications for performing a chest radiograph according to French clinical practice guidelines are summarized in Appendix 2.

### Clinical and Laboratory Data

For each patient, we collected the following information: age, sex, risk factors for invasive pneumococcal disease (e.g., sickle cell disease; complete list in Appendix 3) (18), radiological and laboratory test results, and hospital admission or discharge. For all patients, laboratory results were collected from the initial routine blood analysis, if any. Potential predictors of BC result included complete blood count (notably WBC and ANC), and serum CRP and PCT levels.

### Bacteremia

The outcome of our study was bacteremia, defined by the positivity of BC. BC was performed according to local routines, and no attempt was made to standardize procedures, notably the volume of blood drawn and the techniques used for culture. BC that grew a contaminant (i.e., coagulase-negative *Staphylococcus*, a diphtheroid, *Propionibacterium, Bacillus* species, and *Micrococcus*) were considered negative. Microbiologists assessing the results of BC were not strictly blinded to clinical, imaging, and laboratory information.

### Statistical Analysis

First, we performed a descriptive analysis of participant characteristics and BC results. Proportions were compared using the Chi-Square or Fisher exact test, as appropriate; trends in proportions were assessed using the Cochran-Armitage test. Second, for univariate analysis, we used the non-parametric Mann-Whitney *U*-test to compare the distribution of the four potential predictive biomarkers between participants with and without bacteremia. Third, we used ROC analysis to estimate the area under the ROC curve (AUC-ROC) for each biomarker, and then compared AUC-ROCs using the DeLong test (19); this ROC analysis was restricted to participants with all four biomarkers available. Fourth, for the predictor(s) with the best performance on ROC analysis, we calculated sensitivity, specificity, positive and negative likelihood ratios, and diagnostic odds ratio (DOR) estimates at prespecified rounded thresholds, and identified thresholds able to provide sensitivity and specificity estimates of at least 90%. For each biomarker, the “optimal” cutoff was identified as the cutoff that maximized the DOR, a commonly used indicator of overall test performance (20).

In a sensitivity analysis, we investigated the performance of WBC, ANC, CRP, and PCT to predict “pyogenic CAP,” a composite binary outcome defined by laboratory-confirmed bacterial CAP caused by *S. pneumoniae, Staphylococcus aureus*, or *Streptococcus pyogenes* using any microbiological method among pleural and blood culture, antigen detection, and polymerase chain reaction (PCR; including specific PCRs and 16S ribosomal RNA gene PCR and sequencing) (13).

This is a secondary analysis of an existing database; there was no a priori sample size calculation for this study. Statistical analyses involved the use of Stata 15/SE (Stata Corp., College Station, Texas).

## Results

### Participant Characteristics

**Figure 1:**
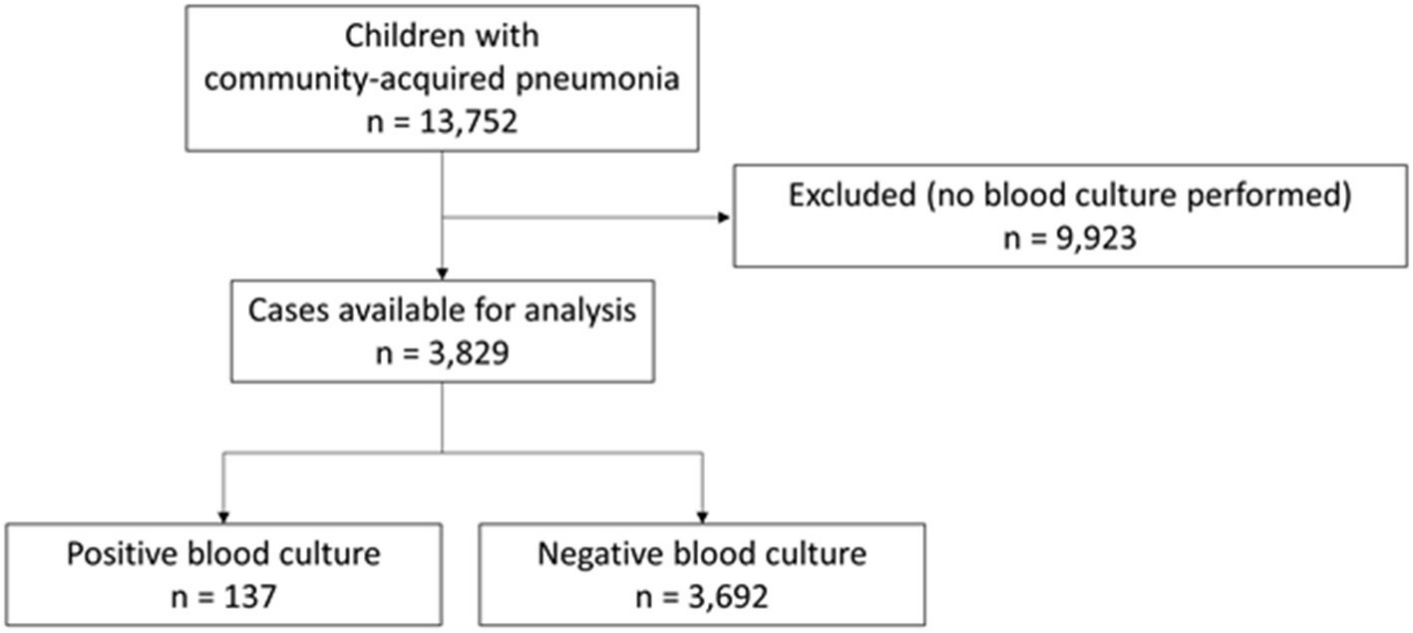
Flow chart of participants through the study.

During the 9-year study period, 13,752 children with CAP were enrolled. In total, 3,829 of these (27.8%) had at least one BC performed and were analyzed (Figure 1). More than 80% of study participants were under 6-year-old, with a mean age of 3.7 years (standard deviation, 3.3); 52.0% were males; 10.7% had a risk factor for invasive pneumococcal disease; 72.1% were admitted to the hospital; 13.7% had pleural effusion (Table 1; Appendix 4). CRP, WBC, ANC, and PCT were measured in 3,751 (98.0%), 3,629 (94.8%), 3,326 (86.9%), and 1,251 (32.7%) study participants, respectively. There was significant variability across centers in the proportion of children in whom a PCT test was ordered (range: 3.4–77.2%, *p* < 0.001; Appendix 5). Children in whom a BC was obtained were more likely to have risk factors of invasive pneumococcal disease (10.7 vs. 7.1%, *p* < 0.001), to be admitted (72.1 vs. 19.3%, *p* < 0.001), and to have pleural effusion (13.7 vs. 2.0%, *p* < 0.001) than children excluded because BC was not performed (Appendix 4).

**Table 1:** Baseline demographic and clinical characteristics of study participants (*N* = 3,829).

**Variable**	**Number**	**%**
**Age, y**		
<2	1,495	39
2-5	1,593	41.6
6-15	741	19.4
Males^*^	1,990	52
Risk factor(s) of invasive pneumococcal disease^**^	410	10.7
Hospital admission^***^	2,762	72.1
**Pleural effusion on initial chest radiograph**	526	13.7

*Missing data for:*

* *50*,

** *89*,

*** *17 children, respectively. See details about risk factors of invasive pneumococcal disease in Appendix 3*.

### Blood Culture Results

**Figure 2:**
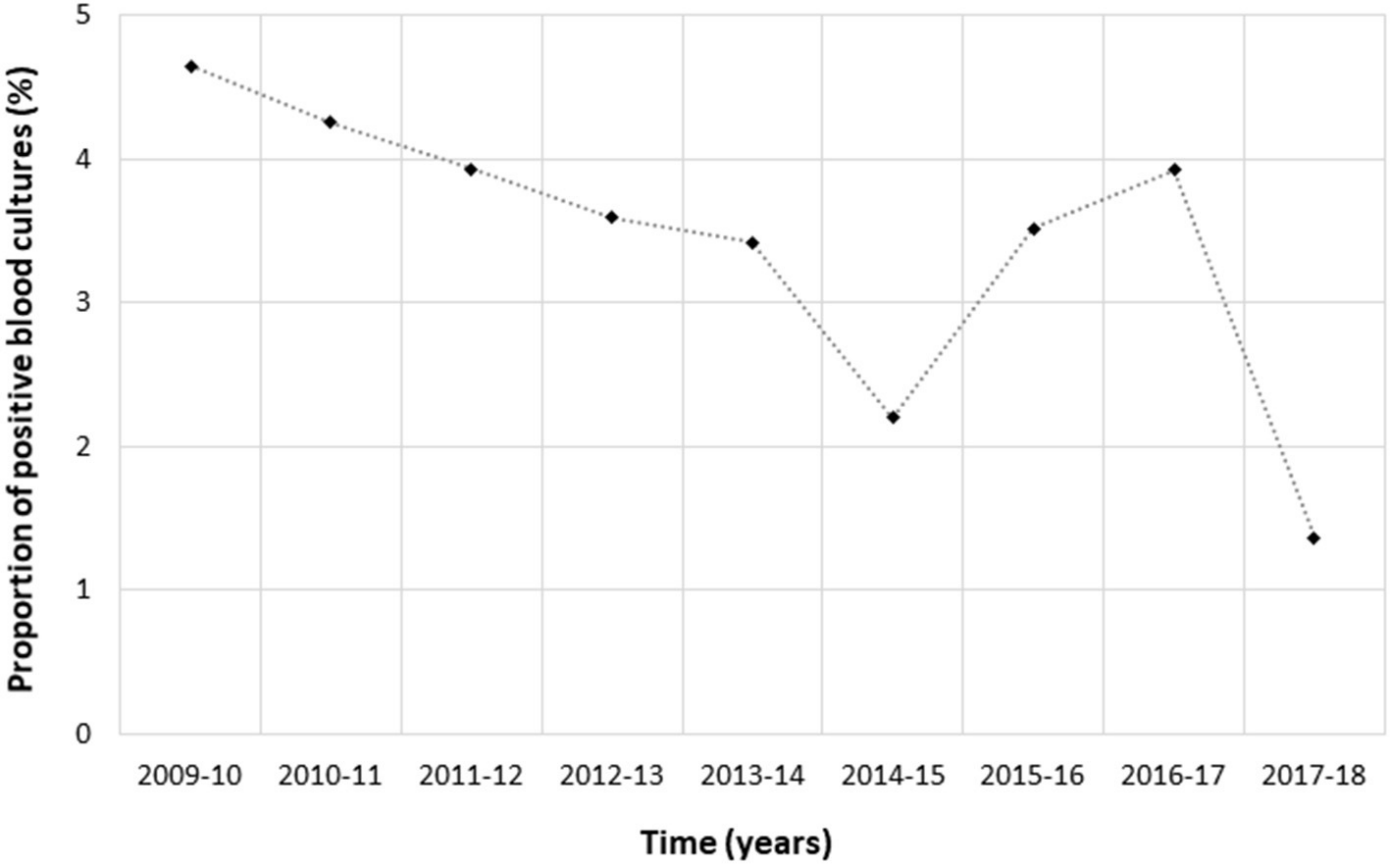
Proportion of positive blood cultures over time (*N* = 3,829).

In total, 137 children out of 3,829 (3.6%) had bacteremia (Figure 2). *S. pneumoniae* was the most prevalent bacterial species isolated in BC (*n* = 107, 78.1%), followed by *S. aureus* (*n* = 18, 13.1%), and *S. pyogenes* (*n* = 4, 2.9%); 2 BC were positive for *Haemophilus influenzae*; 1 was positive for *Pseudomonas aeruginosa*; 1 was positive for *Klebsiella pneumoniae*, 1 was positive for *Escherichia coli*; 3 BC were reported as positive but with missing data for the isolated bacterial species (Appendix 6). The proportion of *S. pneumoniae* among positive BC decreased over time (χ^2^ for trend, *p* = 0.015). Among the 3,829 participants in whom a blood culture was done, the proportion of children with bacteremia was not significantly different in those with and without biomarkers performed (Appendix 7).

### Distribution of Predictors by Blood Culture Result

We did not find any significant difference in WBC levels depending on BC result (median 17,005/mm^3^ (interquartile range 9,575–23,248) in BC-positives vs. 14,400/mm^3^ (IQR 9,000–21,260) in BC-negatives, *p* = 0.07). ANC, CRP and PCT levels were significantly higher in patients with bacteremia compared to patients with negative BC: median values were 12,256/mm^3^ (IQR 7,376–17,869) vs. 9,251/mm^3^ (IQR 5,000–15,287) for ANC (*p* = 0.002), 223 mg/L (IQR 94–316) vs. 72 mg/L (IQR 29–183) for CRP (*p* < 0.001), and 8.6 ng/mL (2.7–21.6) vs. 1.0 ng/mL (0.3–5.4) for PCT (*p* < 0.001; Table 2), respectively. Rates of positive BC increased with increasing levels of CRP and PCT (*p* < 0.001 for both; Appendix 8).

**Table 2:** Comparison of biomarkers among participants with and without bacteremia: univariate analysis.

		**Bacteremia**		**No bacteremia**		
**Biomarker**	***N***	**Median**	**IQR**	**Median**	**IQR**	***p***
WBC (/mm^3^)	3,629	17,005	9,575–23,248	14,400	9,000–21,260	0.07
ANC (/mm^3^)	3,326	12,256	7,376–17,869	9,251	5,000–15,287	0.002
CRP (mg/L)	3,751	223	94–316	72	29–183	<0.001
PCT (ng/mL)	1,251	8.6	2.7–21.6	1	0.3–5.4	<0.001

*IQR, interquartile range; WBC, white blood cells; ANC, absolute neutrophils count; CRP, C-reactive protein; PCT, procalcitonin*.

### ROC Analysis

**Figure 3:**
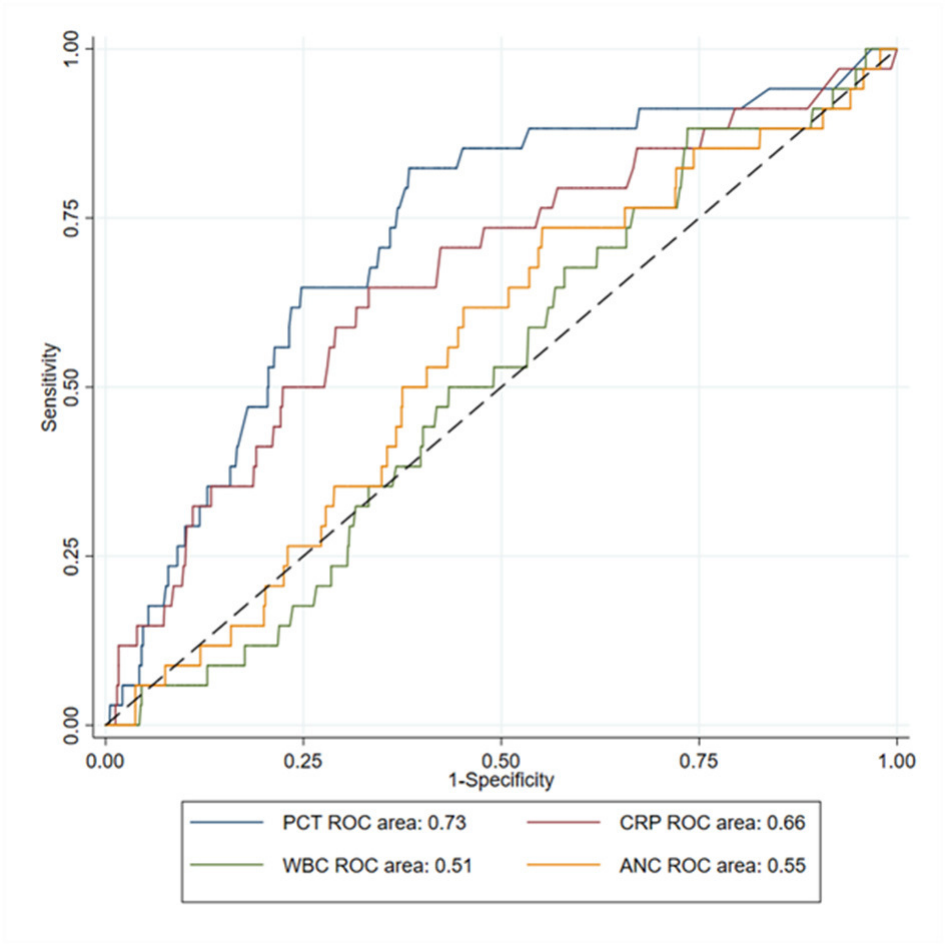
ROC curves of white blood cells (WBC), absolute neutrophils count (ANC), C-reactive protein (CRP) and procalcitonin (PCT) to predict bacteremia in children with community-acquired pneumonia (*N* = 1,069).

Among the 1,069 children with all four biomarkers available, 34 (3.2%) had bacteremia, with a majority of *S. pneumoniae* (*n* = 27, 79.4%). The biomarker with the highest AUC-ROC was PCT, with a value of 0.73 (95%CI 0.64–0.82). It was significantly higher than AUC-ROC of WBC (0.51 [0.43–0.60], *p* < 0.001), and than AUC-ROC of ANC (0.55 [0.46–0.64], *p* = 0.01). It was also higher than AUC-ROC of CRP, but the difference was not statistically significant (0.66 [0.56–0.76], *p* = 0.21; Figure 3).

### Diagnostic Accuracy of CRP and PCT at Predefined Thresholds

The detailed diagnostic accuracy of CRP and PCT in predicting bacteremia is presented in Table 3; Appendix 9. The highest CRP threshold that provided a sensitivity of at least 90% was of 30 mg/L (sensitivity 91.8 [85.8–95.8%], for a specificity of 25.4 [24.0–26.9%]). The lowest CRP threshold that provided a specificity of at least 90% was 300 mg/L (sensitivity 31.3 [23.6–39.9%], for a specificity of 90.6 [89.6–91.5%]). The CRP threshold that maximized the DOR was 50 mg/L (DOR 4.40 [2.65–7.31]).

**Table 3:** Diagnostic accuracy of C-reactive protein (*N* = 3,751) and procalcitonin (*N* = 1,251) to predict bacteremia.

	**Sensitivity, % (95%CI)**	**Specificity, % (95%CI)**	**PLR (95%CI)**	**NLR (95%CI)**	**DOR (95%CI)**
**CRP threshold (mg/L)**					
20	93.3 (87.6–96.9)	18.0 (16.8–19.3)	1.14 (1.08–1.19)	0.37 (0.20–0.70)	3.05 (1.56–5.95)
50	87.3 (80.5–92.4)	39.0 (37.4–40.6)	1.43 (1.34–1.53)	0.33 (0.21–0.51)	4.40 (2.65–7.31)
100	74.6 (66.4–81.7)	58.3 (56.7–59.9)	1.79 (1.61–1.99)	0.43 (0.32–0.58)	4.12 (2.78–6.10)
**PCT threshold (ng/mL)**					
0.5	89.5 (75.2–97.1)	37.7 (34.9–40.5)	1.44 (1.28–1.61)	0.28 (0.11–0.71)	5.14 (1.89–13.98)
2	81.6 (65.7–92.3)	59.1 (56.3–61.9)	2.00 (1.69–2.35)	0.31 (0.16–0.61)	6.40 (2.86–14.34)
4	65.8 (48.6–80.4)	69.6 (66.9–72.2)	2.16 (1.69–2.76)	0.49 (0.32–0.77)	4.40 (2.25–8.59)

*CRP, C-reactive protein; PCT, procalcitonin; PLR, positive likelihood ratio; NLR, negative likelihood ratio; DOR, diagnostic odds ratio*.

The highest PCT threshold that provided a sensitivity of at least 90% was of 0.25 ng/mL (sensitivity 92.1 [78.6–98.3%], for a specificity of 23.4 [21.1–25.9%]). The lowest PCT threshold that provided a specificity of at least 90% was 20 ng/mL (sensitivity 28.9 [15.4–45.9%], for a specificity of 90.6 [88.8–92.2%]). The PCT threshold that maximized the DOR was 2 ng/mL (DOR 6.40 [2.86–14.34]).

### Sensitivity Analysis

In the sensitivity analysis investigating pyogenic CAP, 1,466 participants had all biomarkers available. A pyogenic cause of CAP was identified in 74 (5.0%) cases (*S. pneumoniae, n* = 51 [68.9%]; *S. aureus, n* = 13 [17.6%]; *S. pyogenes, n* = 11 [14.9%]). The biomarker with the highest AUC-ROC was CRP, with a value of 0.79 (95%CI 0.74–0.84). It was significantly higher than AUC-ROC of WBC (0.60 [0.53–0.66], *p* < 0.001), and than AUC-ROC of ANC (0.64 [0.58–0.71], *p* < 0.001). It was also higher than AUC-ROC of PCT, but the difference was not statistically significant (0.76 [0.70–0.82], *p* = 0.98; Appendix 10).

## Discussion

### Main Findings

In pediatric CAP, distinguishing viral from bacterial etiology is particularly challenging. Furthermore, among bacterial etiologies, bacteremic pneumonia represents a small subset of bacterial pneumonia, but probably the most severe cases. In this large prospective multicenter study of children with CAP, we investigated whether routinely available biomarkers such as WBC, ANC, CRP, and PCT were able to predict bacteremia. Bacteremia was present in <5% of children, mostly with *S. pneumoniae*. Our results show that PCT and CRP are the most accurate routinely available predictors of bacteremia in children with CAP. Even if PCT seemed to have better classification properties than other biomarkers (i.e., with the highest AUC-ROC) it was not significantly better than CRP. Results were comparable in a sensitivity analysis investigating pyogenic CAP as an alternative outcome.

### Implications

Our study adds to previous evidence suggesting that PCT might be a reliable routinely available biomarker to predict bacterial etiology in children with CAP, especially pneumococcal etiology (12). Our results are in line with previous observational studies and also confirm that PCT might perform better than other routinely available biomarkers for predicting bacteremia in children with CAP (10). Furthermore, PCT seems the best biomarker for predicting the severity of CAP, which includes bacteremia, but also empyema and pleural effusion (11). Cohen et al. found that PCT was the best routinely available biomarker for predicting clinical response to beta-lactam treatment in children with CAP (21), again suggesting that PCT might be a surrogate marker of pneumococcal etiology. Our results confirm the potential usefulness of CRP and PCT in estimating the efficacy of PCVs among children (13, 14), and determining the eligibility of children for clinical trials of CAP, as has been proposed for adults (22). The advantages of CRP compared to PCT are the availability of rapid point-of-care tests able to provide a result in ≤5 min (23), and its low cost.

As in previous evaluations (24), cases of bacteremic CAP were rare in our study, representing ≤4% of study participants in whom a BC was performed. Even when extended diagnostic workup was conducted, pyogenic CAP remained documented in only 5% of children. An explanation could be that the burden of invasive pneumococcal disease significantly decreased over the past decade because of the implementation of PCVs (14, 25, 26), which were introduced in France in 2002 (7-valent PCV) and 2010 (13-valent PCV).

In adults, PCT predicts pneumococcal bacteremia in patients with CAP (27). Also, Christ-Crain et al. designed a treatment algorithm relying on the high negative predictive value of PCT to rule-out bacterial CAP (28). In a recent individual patient data meta-analysis, including 26 trials, this algorithm proved able to reduce antibiotic prescription rates and to shorten antibiotic exposure without any increase in mortality (29). In children, the available evidence to support PCT-guided treatment algorithms is much weaker and includes only two randomized controlled trials. Using a 0.25 ng/mL PCT threshold, Esposito et al. found results comparable to those from adult trials, with a significant reduction of both antibiotic treatment rate and duration (30). Conversely, Baer et al. showed no significant reduction in antibiotic treatment rates (31). A reason for such a discrepancy could be that PCT treatment thresholds defined in adult studies (0.25 ng/mL) might be too low for decision-making in children with CAP. For example, in our study, using a 2 ng/mL threshold maximized the DOR of PCT for predicting bacteremia (DOR 6.40, 95%CI 2.86–14.34), but this association remains too weak to recommend PCT as a standalone test for treatment decisions in children with CAP. The aim of this study was to assess the association between biomarkers and bacteremic pneumonia. As in other diseases such as meningitis, it might be relevant to combine PCT with clinical information (such as immunization status, risk factors for pneumococcal infection, duration of symptoms, systemic and respiratory symptoms, and age) and other biomarkers into clinical decision rules (32).

### Strengths and Limitations

The strengths of our study are the prospective and multicenter enrollment and the large sample size. We benefit from a high external validity regarding the occurrence of bacteremia (3.6%) and the prevalence of *S. pneumoniae* (78.1%), which are close to that from others studies, respectively 2.5 and 78% in Neuman's North-American study (33), and 5.2 and 76.7% in Tam's meta-analysis (24).

Our study has several limitations. First, <30% of children with CAP had a BC performed. Children in whom a BC is obtained might have more severe forms of CAP (Appendix 4), and we cannot exclude selection bias. In this study, the diagnostic accuracy of CRP and PCT might have been overestimated as a result of this selection bias. This also means that our findings are more likely to apply to inpatients compared to outpatients. Furthermore, positive BC is far from a “gold standard” to evaluate the cause of CAP. Moreover, less than a third of included participants in whom a BC was performed had all four biomarkers available for analysis. While CRP measurements were obtained in 98% of study participants, PCT levels were assessed in only 33% of cases, showing that PCT is not obtained routinely. Also, we opted for a complete case analysis which led to many patient exclusions, notably because PCT was done only in 1,251 out of 3,829 (32.7%) children in whom a blood culture was performed. This was the only way to ensure patient comparability throughout the analyses, but it may have introduced bias toward more severe patients and some participating centers. Another limitation is that we lack the exact timing of biomarker measurements. In the case of early measurements (i.e., <24 h of infection onset), PCT may perform better because its concentration may peak quicker than that of CRP (34). Finally, PCT had the highest AUC-ROC, but we may have lacked statistical power to find a significant difference between the predictive ability of PCT and CRP.

## Conclusions

In summary, CRP and PCT are the best routinely available biomarkers to predict bacteremia in children with CAP. Such biomarkers should be considered when developing new strategies for treatment decisions in children with CAP. We also recommend using CRP or PCT in studies monitoring the epidemiology of CAP and the impact of PCVs.

## Author's Note

Preliminary study results were presented in part at the 7th Congress of the European Academy of Paediatric Societies, Paris, France, November 2018.

## Data Availability Statement

Data underlying our findings are available upon request to ACTIV, and requests can be directed to CL, corinne.levy@activ-france.fr.

## Ethics Statement

The studies involving human participants were reviewed and approved by the Ethics Committee of Robert Debré Hospital. Written informed consent to participate in this study was provided by the participants' legal guardian/next of kin.

## Pneumonia Study Group

**Collaborators (investigators from the Pneumonia Study Group):** François Angoulvant, Department of General Pediatrics, AP-HP, Hôpital Robert Debré, Université de Paris, Paris, France; Yves Gillet, Pediatric Emergency Department, Hospices Civils de Lyon, Hôpital Femme-Mère-Enfant, Lyon, France; Christèle Gras-Le Guen, Department of Pediatrics, Centre Hospitalier Universitaire de Nantes, Nantes, France; Isabelle Hau, Department of Pediatrics, Centre Hospitalier Intercommunal de Créteil, Créteil, France; Laure Hees, Pediatric Emergency Department, Hospices Civils de Lyon, Hôpital Femme-Mère-Enfant, Lyon, France; Elise Launay, Department of Pediatrics, Centre Hospitalier Universitaire de Nantes, Nantes, France; Mathie Lorrot, Department of General Pediatrics, Assistance Publique-Hôpitaux de Paris, Hôpital Armand Trousseau, Université Sorbonne Paris Cité, Paris, France; Fouad Madhi, Department of Pediatrics, Centre Hospitalier Intercommunal de Créteil, Créteil, France; Alain Martinot, Univ. Lille, CHU Lille, Pediatric Emergency Unit and Infectious Diseases, Lille, France; Naim Ouldali, Department of General Pediatrics, AP-HP, Hôpital Robert Debré, Université de Paris, Paris, France.

## Author Contributions

JC: had full access to all of the data in the study and takes responsibility for the integrity of the data and the accuracy of the data analysis. DD, JC, MC, CL, and RC: concept and design, and drafting of the manuscript. DD, JC, and MC: statistical analysis. CL and RC: obtained funding. JC, MC, CL, and RC: supervision. All authors: acquisition, analysis, or interpretation of data, and critical revision of the manuscript for important intellectual content.

## Funding

The ACTIV network received funding from Pfizer for this study. The funder had no role in study design, data collection and analysis, or preparation of the manuscript.

## Conflict of Interest

The authors declare that the research was conducted in the absence of any commercial or financial relationships that could be construed as a potential conflict of interest.

## Publisher's Note

All claims expressed in this article are solely those of the authors and do not necessarily represent those of their affiliated organizations, or those of the publisher, the editors and the reviewers. Any product that may be evaluated in this article, or claim that may be made by its manufacturer, is not guaranteed or endorsed by the publisher.

## Abbreviations

ACTIV: Association Clinique et Thérapeutique Infantile du Val de Marne
ANC: absolute neutrophils count
AUC-ROC: area under the ROC curve
BC: blood culture
CAP: community-acquired pneumonia
CRP: C-reactive protein
DOR: diagnostic odds ratio
PCT: procalcitonin
PCR: polymerase chain reaction
PCV: pneumococcal conjugate vaccine
WBC: white blood cells
95%CI: 95% confidence interval.
