# Supplementary material for: Diagnostic Accuracy of Routinely Available Biomarkers to Predict Bacteremia in Children With Community-Acquired Pneumonia: A Secondary Analysis of the GPIP/ACTIV Pneumonia Study in France, 2009–2018
Source: Front Pediatr. 2021 Oct 21;9:684628. doi: 10.3389/fped.2021.684628 (PMC8567958; doi:10.3389/fped.2021.684628)
Supplement: Supplementary file 1 [file Data_Sheet_1.docx]

**Diagnostic accuracy of routinely available biomarkers to predict bacteremia in children with community-acquired pneumonia: A secondary analysis of the GPIP/ACTIV pneumonia study in France, 2009-2018**

**APPENDICES**

**Appendix 1. STARD 2015 checklist**

|  | **Section & Topic** | **No** | **Item** | **Reported on page #** |
| --- | --- | --- | --- | --- |
|  |  |  |  |  |
|  | **TITLE OR ABSTRACT** |  |  |  |
|  |  | **1** | Identification as a study of diagnostic accuracy using at least one measure of accuracy (such as sensitivity, specificity, predictive values, or AUC) | Abstract |
|  | **ABSTRACT** |  |  |  |
|  |  | **2** | Structured summary of study design, methods, results, and conclusions  (for specific guidance, see STARD for Abstracts) | Abstract |
|  | **INTRODUCTION** |  |  |  |
|  |  | **3** | Scientific and clinical background, including the intended use and clinical role of the index test | 5 |
|  |  | **4** | Study objectives and hypotheses | 5 |
|  | **METHODS** |  |  |  |
|  | *Study design* | **5** | Whether data collection was planned before the index test and reference standard  were performed (prospective study) or after (retrospective study) | 6 |
|  | *Participants* | **6** | Eligibility criteria | 6 |
|  |  | **7** | On what basis potentially eligible participants were identified  (such as symptoms, results from previous tests, inclusion in registry) | 6 |
|  |  | **8** | Where and when potentially eligible participants were identified (setting, location and dates) | 6 |
|  |  | **9** | Whether participants formed a consecutive, random or convenience series | 6 |
|  | *Test methods* | **10a** | Index test, in sufficient detail to allow replication | 7 |
|  |  | **10b** | Reference standard, in sufficient detail to allow replication | 7 |
|  |  | **11** | Rationale for choosing the reference standard (if alternatives exist) | 7 |
|  |  | **12a** | Definition of and rationale for test positivity cut-offs or result categories  of the index test, distinguishing pre-specified from exploratory | 8 |
|  |  | **12b** | Definition of and rationale for test positivity cut-offs or result categories  of the reference standard, distinguishing pre-specified from exploratory | N/A |
|  |  | **13a** | Whether clinical information and reference standard results were available  to the performers/readers of the index test | N/A |
|  |  | **13b** | Whether clinical information and index test results were available  to the assessors of the reference standard | 7 |
|  | *Analysis* | **14** | Methods for estimating or comparing measures of diagnostic accuracy | 7 |
|  |  | **15** | How indeterminate index test or reference standard results were handled | 7 |
|  |  | **16** | How missing data on the index test and reference standard were handled | 7 |
|  |  | **17** | Any analyses of variability in diagnostic accuracy, distinguishing pre-specified from exploratory | N/A |
|  |  | **18** | Intended sample size and how it was determined | 8 |
|  | **RESULTS** |  |  |  |
|  | *Participants* | **19** | Flow of participants, using a diagram | Fig 1 |
|  |  | **20** | Baseline demographic and clinical characteristics of participants | 8-9 |
|  |  | **21a** | Distribution of severity of disease in those with the target condition | 8-9 and Table 1 |
|  |  | **21b** | Distribution of alternative diagnoses in those without the target condition | Not assessed |
|  |  | **22** | Time interval and any clinical interventions between index test and reference standard | Not assessed |
|  | *Test results* | **23** | Cross tabulation of the index test results (or their distribution)  by the results of the reference standard | 9 |
|  |  | **24** | Estimates of diagnostic accuracy and their precision (such as 95% confidence intervals) | 10 and Tables 5-6 |
|  |  | **25** | Any adverse events from performing the index test or the reference standard | N/A |
|  | **DISCUSSION** |  |  |  |
|  |  | **26** | Study limitations, including sources of potential bias, statistical uncertainty, and generalisability | 14 |
|  |  | **27** | Implications for practice, including the intended use and clinical role of the index test | 11-14 |
|  | **OTHER INFORMATION** |  |  |  |
|  |  | **28** | Registration number and name of registry | Not registered |
|  |  | **29** | Where the full study protocol can be accessed | N/A |
|  |  | **30** | Sources of funding and other support; role of funders | Funding statement |
|  |  |  |  |  |

**Appendix 2. Indications for performing a chest radiograph according to French guidelines**

According to French guidelines, indications for performing a chest radiograph in children are as follows:

- child with fever and abnormal auscultation (crackles) and/or tachypnea, except for bronchiolitis;

- fever without source (prolonged or poorly tolerated), notably in infants;

- febrile cough that is prolonged or accompanied with increasing tachypnea;

- child with recurrent pneumonia and/or suspicion of foreign body aspiration;

- diagnostic uncertainty between bronchitis and pneumonia.

*Translated from “Antibiotherapie par voie generale en pratique courante au cours des infections respiratoires basses de l’adulte et de l’enfant. Agence Française de Sécurité Sanitaire des Produits de Santé, octobre 2005. Available at: https://www.infectiologie.com/UserFiles/File/medias/_documents/consensus/2005-infVRB-recos-afssaps.pdf*

**Appendix 3. Risk factors of invasive pneumococcal disease considered in this study**

- Anatomic or functional asplenia
- Homozygous sickle cell disease
- Human immunodeficiency virus infection
- Congenital immunodeficiency or secondary immunodeficiency:
  - chronic renal failure or nephrotic syndrome
  - immunosuppressive therapy or radiotherapy for neoplasia, lymphoma or Hodgkin’s disease, leukemia, organ transplantation
- Cyanotic congenital heart disease, heart failure
- Chronic lung disease (except asthma without long-term corticosteroid therapy)
- Cerebrospinal fluid leakage
- Diabetes
- Be candidate for cochlear implantation or carrier of cochlear implant

**Appendix 4. Comparison of included and excluded patients**

|  | | **Included (N=3,829)** | | **Excluded (N=9,923)** | |  |
| --- | --- | --- | --- | --- | --- | --- |
| **Variable** | | **Number** | **%** | **Number** | **%** | ***p*** |
| **Sex** | |  |  |  |  |  |
|  | Male | 1,990 | 52.0 | 5,254 | 53.0 | 0.042 |
|  | Female | 1,789 | 46.7 | 4,584 | 46.2 |  |
|  | Missing | 50 | 1.3 | 85 | 0.9 |  |
| **Age, y** | |  |  |  |  |  |
|  | 0-2 | 1,495 | 39.0 | 3,594 | 36.2 | < 0.001 |
|  | 2-6 | 1,593 | 41.6 | 3,990 | 40.2 |  |
|  | 6-15 | 741 | 19.4 | 2,339 | 23.57 |  |
| **Risk factor(s) of invasive pneumococcal disease** | |  |  |  |  |  |
|  | Yes | 410 | 10.7 | 702 | 7.1 | < 0.001 |
|  | No | 3,330 | 87.0 | 9,022 | 90.9 |  |
|  | Missing | 89 | 2.3 | 199 | 2.0 |  |
| **Hospital admission** | |  |  |  |  |  |
|  | Yes | 2,762 | 72.1 | 1,913 | 19.3 | < 0.001 |
|  | No | 1,050 | 27.4 | 7,964 | 80.3 |  |
|  | Missing | 17 | 0.5 | 46 | 0.5 |  |
| **Pleural effusion on initial chest radiograph** | |  |  |  |  |  |
|  | Yes | 526 | 13.7 | 202 | 2.0 | < 0.001 |
|  | No | 3,303 | 86.3 | 9,721 | 98.0 |  |

**Appendix 5. Breakdown of PCT ordering across centers (N=3,829)**

|  | **PCT performed** | |
| --- | --- | --- |
| **Center** | **n/N** | % |
| Robert-Debré | 451/953 | 47.3 |
| Kremlin Bicêtre | 46/115 | 40.0 |
| Versailles | 157/352 | 44.6 |
| Lyon | 26/682 | 3.8 |
| Lille | 137/268 | 51.1 |
| Créteil | 23/681 | 3.4 |
| Nantes | 196/254 | 77.2 |
| Marseille | 215/524 | 41.0 |
| TOTAL | 1,251/3,829 | 32.7 |

**Appendix 6. Results of blood cultures in children with community-acquired pneumonia (N=3,829)**

| **Variable** | | **2009-10** | **2010-11** | **2011-12** | **2012-13** | **2013-14** | **2014-15** | **2015-16** | **2016-17** | **2017-18** | **Total** |
| --- | --- | --- | --- | --- | --- | --- | --- | --- | --- | --- | --- |
| **Bacterial species, N (%)** | |  |  |  |  |  |  |  |  |  |  |
|  | *Streptococcus pneumoniae* | 28 (90.3) | 19 (79.2) | 17 (94.4) | 10 (76.9) | 5 (41.7) | 6 (75.0) | 10 (83.3) | 9 (64.3) | 3 (60.0) | 107 (78.1)* |
|  | *Staphylococcus aureus* | 3 (9.7) | 5 (20.8) | 0 (0) | 1 (7.7) | 5 (41.7) | 2 (25.0) | 0 (0) | 2 (14.3) | 0 (0) | 18 (13.1) |
|  | *Streptococcus pyogenes* | 0 (0) | 0 (0) | 1 (5.6) | 1 (7.7) | 0 (0) | 0 (0) | 0 (0) | 1 (7.1) | 1 (20.0) | 4 (2.9) |
|  | *Haemophilus influenzae* | 0 (0) | 0 (0) | 0 (0) | 0 (0) | 0 (0) | 0 (0) | 1 (8.3) | 1 (7.1) | 0 (0) | 2 (1.5) |
|  | *Pseudomonas aeruginosa* | 0 (0) | 0 (0) | 0 (0) | 0 (0) | 1 (8.3) | 0 (0) | 0 (0) | 0 (0) | 0 (0) | 1 (0.7) |
|  | *Klebsiella pneumoniae* | 0 (0) | 0 (0) | 0 (0) | 1 (7.7) | 0 (0) | 0 (0) | 0 (0) | 0 (0) | 0 (0) | 1 (0.7) |
|  | *Escherichia coli* | 0 (0) | 0 (0) | 0 (0) | 0 (0) | 0 (0) | 0 (0) | 0 (0) | 1 (7.1) | 0 (0) | 1 (0.7) |
|  | Unspecified | 0 (0) | 0 (0) | 0 (0) | 0 (0) | 1 (8.3) | 0 (0) | 1 (8.3) | 0 (0) | 1 (20.0) | 3 (2.2) |
| **Positive blood cultures, N** | | 31 (100) | 24 (100) | 18 (100) | 13 (100) | 12 (100) | 8 (100) | 12 (100) | 14 (100) | 5 (100) | 137 (100) |
| **Number of blood cultures performed** | | 667 | 564 | 458 | 362 | 351 | 363 | 341 | 357 | 366 | 3,829 |
| **Proportion of positive blood cultures** | | 4.6 | 4.3 | 3.9 | 3.6 | 3.4 | 2.2 | 3.5 | 3.9 | 1.4 | 3.6 |

*Test for trend of % *S. pneumoniae* among positive blood cultures over time: *χ^2^* = 5.92, *p* = 0.015.

**Appendix 7. Proportion of children with bacteremia in those with and without biomarkers performed (N=3,829)**

|  |  | **Bacteremia** | | **No bacteremia** | |  |
| --- | --- | --- | --- | --- | --- | --- |
| **Biomarker** | **N** | **N** | **%** | **N** | **%** | ***p*** |
| **WBC** |  |  |  |  |  |  |
| - performed | 3,629 | 132 | 3.6 | 3,497 | 96.4 | 0.399 |
| - not performed | 200 | 5 | 2.5 | 195 | 97.5 |  |
| **ANC** |  |  |  |  |  |  |
| - performed | 3,326 | 119 | 3.6 | 3,207 | 96.4 | 0.999 |
| - not performed | 503 | 18 | 3.6 | 485 | 96.4 |  |
| **CRP** |  |  |  |  |  |  |
| - performed | 3,751 | 134 | 3.6 | 3,617 | 96.4 | 0.897 |
| - not performed | 78 | 3 | 3.9 | 75 | 96.1 |  |
| **PCT** |  |  |  |  |  |  |
| - performed | 1,251 | 38 | 3.0 | 1,213 | 97.0 | 0.210 |
| - not performed | 2,578 | 99 | 3.8 | 2,479 | 96.2 |  |

**Appendix 8. Rates of positive blood culture by levels of C-reactive protein and procalcitonin**

| **Biomarker** | | **Bacteremia, n/N (%)** | ***p*** |
| --- | --- | --- | --- |
| CRP (mg/L) | |  |  |
|  | < 20 | 9/660 (1.4) | <0.001 |
|  | 20 – 100 | 25/1,484 (1.7) |  |
|  | ≥ 100 | 100/1,607 (6.2) |  |
|  | Total | 134/3,751 (3.6) |  |
| PCT (ng/mL) | |  |  |
|  | < 0.5 | 4/461 (0.9) | <0.001 |
|  | 0.5 – 4.0 | 9/396 (2.3) |  |
|  | ≥ 4.0 | 25/394 (6.4) |  |
|  | Total | 38/1,251 (3.0) |  |

CRP, C-reactive protein; PCT, procalcitonin.

**Appendix 9. Detailed diagnostic accuracy of C-reactive protein (CRP) and procalcitonin (PCT) in predicting bacteremia**

|  | | **Sensitivity, % (95%CI)** | **Specificity, % (95%CI)** | **PLR (95%CI)** | **NLR (95%CI)** | **DOR (95%CI)** |
| --- | --- | --- | --- | --- | --- | --- |
| **CRP threshold (mg/L)** | |  |  |  |  |  |
|  | 5 | 97.8 (93.6 – 99.5) | 2.0 (1.6 – 2.6) | 1.00 (0.97 – 1.02) | 1.09 (0.35 – 3.43) | 0.91 (0.30 – 2.77) |
|  | 10 | 97.8 (93.6 – 99.5) | 6.5 (5.7 – 7.3) | 1.05 (1.02 – 1.07) | 0.34 (0.11 – 1.06) | 3.03 (1.01 – 9.09) |
|  | 20 | 93.3 (87.6 – 96.9) | 18.0 (16.8 – 19.3) | 1.14 (1.08 – 1.19) | 0.37 (0.20 – 0.70) | 3.05 (1.56 – 5.95) |
|  | 30 | 91.8 (85.8 – 95.8) | 25.4 (24.0 – 26.9) | 1.23 (1.17 – 1.30) | 0.32 (0.18 – 0.57) | 3.81 (2.07 – 7.02) |
|  | 50 | 87.3 (80.5 – 92.4) | 39.0 (37.4 – 40.6) | 1.43 (1.34 – 1.53) | 0.33 ( 0.21 – 0.51) | 4.40 (2.65 – 7.31) |
|  | 100 | 74.6 (66.4 – 81.7) | 58.3 (56.7 – 59.9) | 1.79 (1.61 – 1.99) | 0.43 (0.32 – 0.58) | 4.12 (2.78 – 6.10) |
|  | 150 | 63.4 (54.7 – 71.6) | 69.4 (67.9 – 70.9) | 2.07 (1.81 – 2.38) | 0.53 (0.42 – 0.66) | 3.94 (2.76 – 5.63) |
|  | 200 | 55.2 (46.6 – 63.8) | 77.6 (76.2 – 79.0) | 2.47 (2.09 – 2.91) | 0.58 (0.48 – 0.70) | 4.27 (3.02 – 6.05) |
|  | 250 | 44.0 (35.5 – 52.9) | 84.4 (83.2 – 85.6) | 2.83 (2.30 – 3.47) | 0.66 (0.57 – 0.77) | 4.27 (3.00 – 6.06) |
|  | 300 | 31.3 (23.6 – 39.9) | 90.6 (89.6 – 91.5) | 3.32 (2.54 – 4.36) | 0.76 (0.68 – 0.85) | 4.39 (3.00 – 6.41) |
| **PCT threshold (ng/mL)** | |  |  |  |  |  |
|  | 0.25 | 92.1 (78.6 – 98.3) | 23.4 (21.1 – 25.9) | 1.20 (1.09 – 1.33) | 0.34 (0.11 – 1.00) | 3.57 (1.15 – 11.00) |
|  | 0.5 | 89.5 (75.2 – 97.1) | 37.7 (34.9 – 40.5) | 1.44 (1.28 – 1.61) | 0.28 (0.11 – 0.71) | 5.14 (1.89 – 13.98) |
|  | 1.0 | 84.2 (68.7 – 94.0) | 49.5 (46.6 – 52.3) | 1.67 (1.44 – 1.93) | 0.32 (0.15 – 0.67) | 5.22 (2.22 – 12.25) |
|  | 2.0 | 81.6 (65.7 – 92.3) | 59.1 (56.3 – 61.9) | 2.00 (1.69 – 2.35) | 0.31 (0.16 – 0.61) | 6.40 (2.86 – 14.34) |
|  | 3.0 | 71.1 (54.1 – 84.6) | 65.9 (63.1 – 68.5) | 2.08 (1.67 – 2.59) | 0.44 (0.27 – 0.72) | 4.74 (2.36 – 9.52) |
|  | 4.0 | 65.8 (48.6 – 80.4) | 69.6 (66.9 – 72.2) | 2.16 (1.69 – 2.76) | 0.49 (0.32 – 0.77) | 4.40 (2.25 – 8.59) |
|  | 5.0 | 65.8 (48.6 – 80.4) | 73.1 (70.5 – 75.6) | 2.45 (1.91 – 3.13) | 0.47 (0.30 – 0.73) | 5.23 (2.68 – 10.23) |
|  | 10.0 | 47.4 (31.0 – 64.2) | 83.3 (81.0 – 85.3) | 2.83 (1.98 – 4.05) | 0.63 (0.47 – 0.86) | 4.48 (2.35 – 8.53) |
|  | 15.0 | 31.6 (17.5 – 48.7) | 88.4 (86.4 – 90.1) | 2.72 (1.66 – 4.45) | 0.77 (0.62 – 0.96) | 3.51 (1.75 – 7.03) |
|  | 20.0 | 28.9 (15.4 – 45.9) | 90.6 (88.8 – 92.2) | 3.08 (1.82 – 5.22) | 0.78 (0.64 – 0.96) | 3.93 (1.92 – 8.03) |

CRP, C-reactive protein; PCT, procalcitonin; PLR, positive likelihood ratio; NLR, negative likelihood ratio; DOR, diagnostic odds ratio.

**Appendix 10. ROC curves for pyogenic pneumonia (N=1,466)
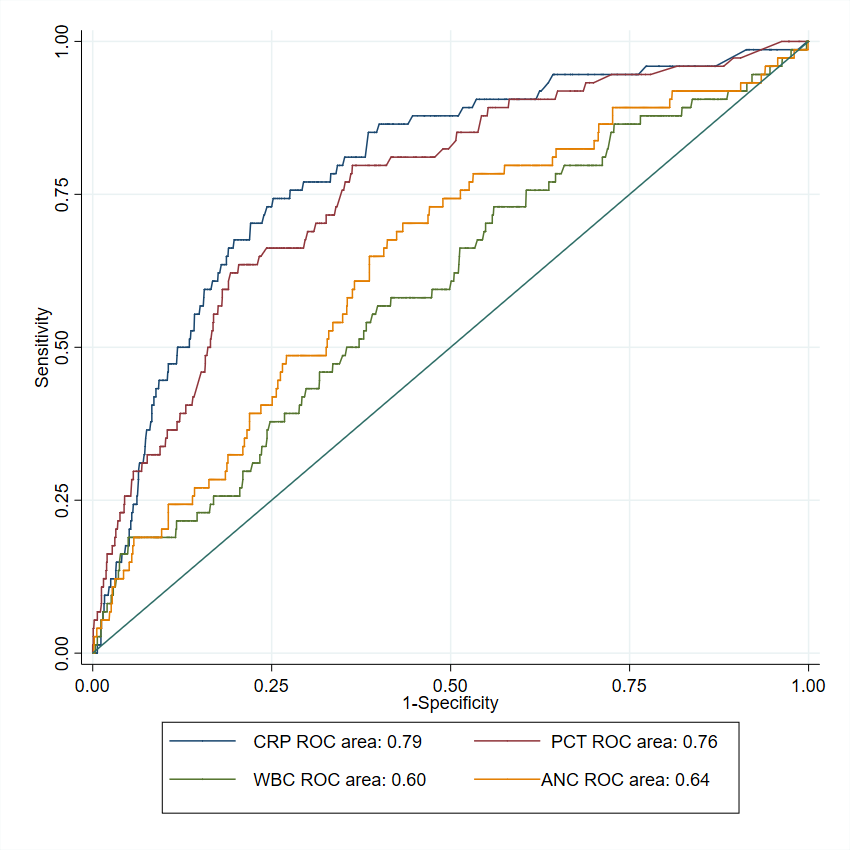
**
